# Broadband photodetection using one-step CVD-fabricated MoS_2_/MoO_2_ microflower/microfiber heterostructures

**DOI:** 10.1038/s41598-022-26185-z

**Published:** 2022-12-21

**Authors:** D. Mouloua, N. S. Rajput, S. Saitzek, K. Kaja, K. Hoummada, M. El Marssi, M. A. El Khakani, M. Jouiad

**Affiliations:** 1 Laboratory of Physics of Condensed Matter, University of Picardie Jules Verne, Scientific Pole, 33 Rue Saint-Leu, 80039 Amiens Cedex 1, France; 2 Institut National de la Recherche Scientifique, Centre-Énergie, Matériaux et Télécommunications, 1650, Blvd, Lionel-Boulet, Varennes, QC J3X-1P7 Canada; 3 Advanced Materials Research Center, Technology Innovation Institute, P.O. Box 9639, Abu Dhabi, United Arab Emirates; 4 UMR 8181, Unité de Catalyse et Chimie du Solide (UCCS), Université d’Artois, CNRS, Centrale Lille, Université de Lille, 62300 Lens, France; 5 Laboratoire National de Métrologie et d’essais (LNE), 29 Av. Roger Hannequin, 78197 Trappes, France; 6 IM2NP, Aix Marseille Université, CNRS, Université de Toulon, 13397 Marseille, France

**Keywords:** Nanoscale materials, Two-dimensional materials

## Abstract

Molybdenum disulfide (MoS_2_) has been combined so far with other photodetecting semiconductors as an enhancing agent owing to its optical and electronic properties. Existing approaches demonstrated MoS_2_-incorporated photodetector devices using complex and costly fabrication processes. Here, we report on simplified one-step on the chemical vapor deposition (CVD) based synthesis of a unique microfiber/microflower MoS_2_-based heterostructure formed by capturing MoO_2_ intermediate material during the CVD process. This particular morphology engenders a material chemical and electronic interplay exalting the heterostructure absorption up to ~ 98% over a large spectral range between 200 and 1500 nm. An arsenal of characterization methods were used to elucidate the properties of these novel heterostructures including Raman spectroscopy, X-ray diffraction, X-ray photoelectron spectrometry, high-resolution transmission and scanning electron microscopies, and Kelvin probe force microscopy. Our findings revealed that the MoS_2_ and the MoO_2_ crystallize in the hexagonal and monoclinic lattices, respectively. The integration of the MoS_2_/MoO_2_ heterostructures into functional photodetectors revealed a strong photoresponse under both standard sun illumination AM1.5G and blue light excitation at 450 nm. Responsivity and detectivity values as high as 0.75 mA W^−1^ and 1.45 × 10^7^ Jones, respectively, were obtained with the lowest light intensity of 20 mW cm^−2^ at only 1 V bias. These results demonstrate the high performances achieved by the unique MoS_2_/MoO_2_ heterostructure for broadband light harvesting and pave the way for their adoption in photodetection applications.

## Introduction

Undeniably, molybdenum disulfide (MoS_2_) has proven to be an excellent material for opto-electronic applications among the family of transition metal dichalcogenides (TMDs) two-dimensional (2D) materials^1^. Its outstanding electrical and optical properties^2–6^ have made MoS_2_ one of the most promising candidates for visible light-driven photodetectors^7^. Nonetheless, carriers’ recombinations in pristine MoS_2_ have limited its photodetecion efficiency, despite its good light absorption performance^8,9^. This has driven a general trend during the last decade combining MoS_2_ with other semiconductors with already known and proven photodetecting capabilities. Efforts done in this direction mainly exploited the high light absorption properties of MoS_2_ to improve the photodetecting performances of the other semiconductor by favoring carriers separation through the creation of a built-in electric field^6–9^. As example, ZnS/MoS_2_ heterostructures have been shown to exhibit a large photodetection capability, where ZnS has served as local electric field generator, achieving an increased optoelectronic performances^10^. The the ultra-violet (UV) detection property of ZnS, combined with the strong visible light absorption of MoS_2_, have led to the fabrication of photodetectors spanning the whole spectrum from UV to the near infrared range. Another strategy consists of using plasmonic metal to create Schottky contact with MoS_2_ for hot electrons injection leading to enhanced optical absorption and good photodetection properties^11–13^. Nonetheless, these approaches adopted so far have merely used MoS_2_ as a supporting agent to catalyze light absorption of other semiconducting photodetector materials. Despite their advantages, these methods still suffer from several challenging limitations to their efficient integration in large scale broadband photodetector applications. Most prominently, fabrication processes of the used semiconductors, such as ZnO or GaN, are highly demanding in terms of resources, time, and cost. Additionally, the resulting functional devices remain restricted to very small dimensions and their efficient photodetection yield is bound to the application of high voltages (i.e. ~ 20 V)^14^. Therefore, innovative solutions to develop large-scale MoS_2_-based structures with high photodetection performances using affordable and efficient fabrication approaches are required to propel the integration of broadband MoS_2_-based photodetectors^15,16^. For this, exploiting MoS_2_-derived semiconductors (such as MoO_2_ and MoO_3_), naturally occurring during fabrication processes, constitutes a novel viable route to develop new heterostructures with enhance photodetecting performances. This is motivated by recently reported results showing a great potential of MoO_2_ as a suitable candidate for optoelectronic applications based heterostructure^17,18^. Recent findings strongly suggest a significant impact of the MoO_2_/MoS_2_ and MoO_3_/MoS_2_ morphology on the increase of optical absorption properties leading to amplified photodetection performances, as found in vertically aligned MoS_2_ needle-like structures^19,20^. Hence, controlling the morphology of MoS_2_ is a key for improving photodetection performances^21,22^. Chemical vapor deposition (CVD) has proven to be one of the most promising fabrication techniques to achieve a controlled growth of MoS_2_^23^, owing to its ease of implementation, low-cost and scalability^24–28^. Nevertheless, this requires the control of multiple parameters including pressure, temperature, heating rate, carrier gas flow rate, substrate, precursors positions, and reaction dwell time. All these processing parameters affect the morphology, crystallinity, and thickness of the MoS_2_ heterostructures, which have been documented elsewhere for the synthesis of high-quality, large-surface-area single and few-layers MoS_2_^29–31^. Interestingly, MoO_2_ is intermediate material created during the MoS_2_ CVD process^32^. Therefore, “capturing” the formation of these materials along with CVD MoS_2_ would enable an innovative approach to create seamless MoS_2_/MoO_2_ heterostructures in a one single step process.

In this study, we present pioneering results on a single step large-scale controlled CVD growth of a unique MoS_2_/MoO_2_ heterostructure showing giant light absorption (i.e. ~ 98%) over the full spectral range from 200 to 1500 nm. The as-grown heterostructures present a special MoO_2_ microflowers/MoS_2_ microfibers morphology, which was found to exhibit a huge specific area along with a strong broadband light absorption spanning from UV to near IR. An arsenal of characterization methods were used to investigate the optical, structural, crystallographic, chemical, electric, and photoelectric properties of the novel heterostructures. We have further integrated the fabricated heterostructure films into a photodetector test configuration to demonstrate their high potential for broadband applications. Applied voltages, one order of magnitude lower that those used for MoS_2_/GaN structures, enabled a comparable photodetectivity for the unique heterostructures morphology.

## Experimental section

### MoS_2_/MoO_2_ synthesis

**Figure 1:**
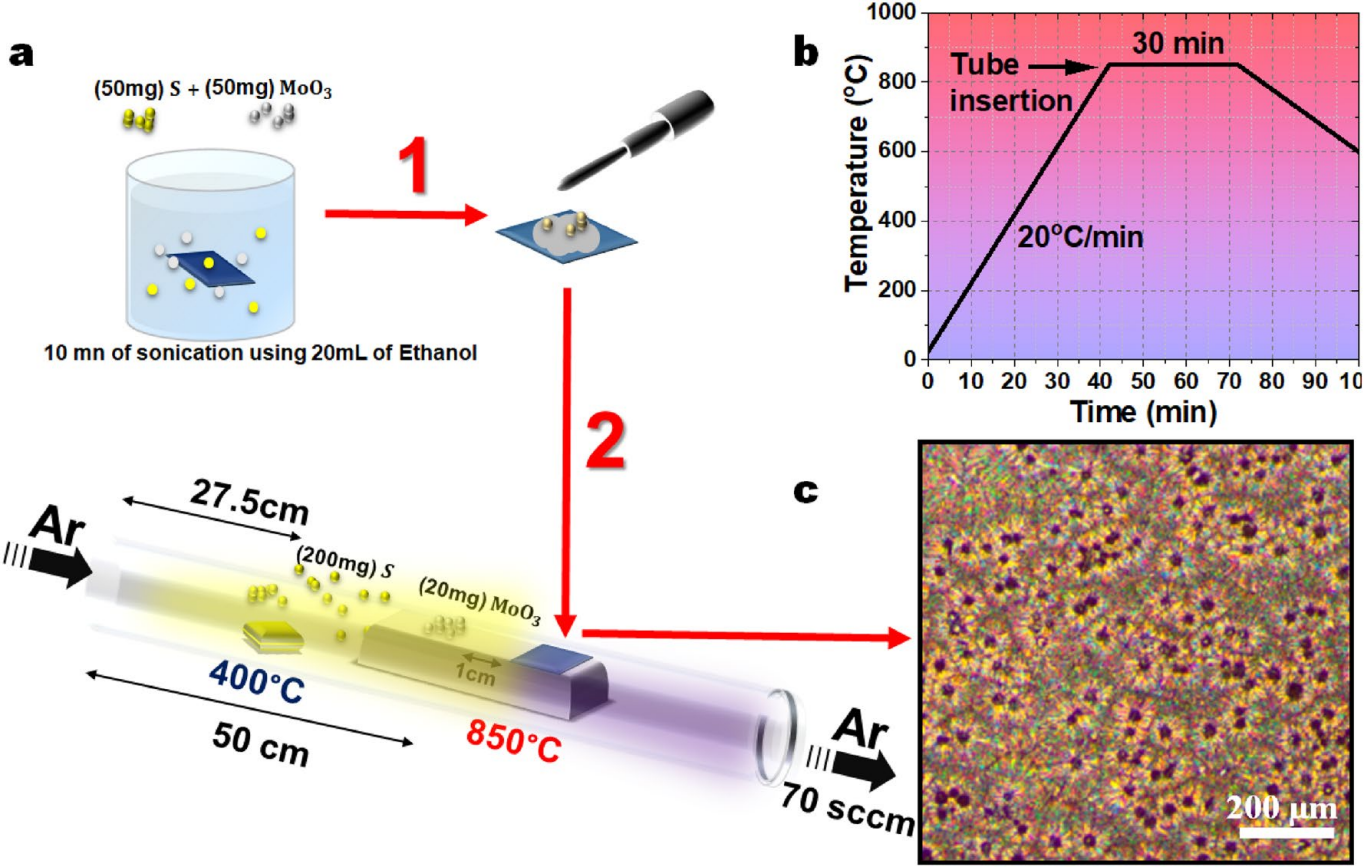
(**a**) CVD process set up of MoS_2_/MoO_2_ heterostructure using a tube furnace. (**b**) The temperature profile used for the synthesis of MoS_2_/MoO_2_ heterostructures. (**c**) Optical image of the typically obtained MoS_2_/MoO_2_ heterostructure film on Si substrate.

The CVD growth conditions were optimized to control the morphology and ratio of the MoS_2_/MoO_2_ in the heterostructure grown on silicon substrates. First, the intrinsic Si substrate was successively cleaned with acetone and ethanol, rinsed with deionized water, and the dried with a nitrogen jet before introducing it into the furnace. Molybdenum trioxide (MoO_3_, 99.99%) and sulfur (S, 99.5%) powders were used as reactant and precursor materials, respectively. The cleaned Si substrate was immersed into a mixture solution consisting of 50 mg of S, 50 mg of MoO_3_, and ethanol. The mixture was kept in an ultrasonicator for 10 min with the Si substrate immersed in it. Then, the substrate was removed from solution and few droplets were added onto its surface before introduction into the horizontal quartz tube of the CVD reactor. A ceramic boat with 200 mg of sulfur was placed upstream in the low-temperature zone of the furnace, 27.5 cm from the flow inlet. Another 2 cm-thick boat was placed face-down downstream in the center of the furnace to exploit its thickness in our growth process. An excess of 20 mg MoO_3_ was added on the top of the boat at the hot zone (50 cm from the flow inlet). The Si substrate (1 cm^2^) was placed on the top of this boat 1 cm from the MoO_3_ powder, as shown in Fig. 1a. Ultra-high purity Ar gas was flown in the furnace at the rate of 70 sccm during the whole growth process. The center of the furnace was heated from room temperature to 850 °C with a rate of 20 °C/min to achieve a non-homogeneous temperature profile inside the quartz tube with an incomplete transformation from MoO_3_ powder to MoS_2_ film (Fig. 1b). The furnace was kept at the growth temperature for 30 min. All syntheses were done at atmospheric pressure. Finally, the furnace was allowed to cool down naturally to room temperature with 70 sccm Ar flow. The optical image of the sample surface is given in Fig. 1c showing the homogeneous deposition.

### Characterization

An Olympus BX51M optical microscope was used to observe the morphology of the MoS_2_/MoO_2_ using bright field mode. Scanning electron microscopy (SEM) (Quanta 200 FEG, ThermofisherScientific) was used to observe the microstructure and examine the nucleation mechanism of our samples growth. The energy-dispersive X-ray spectroscopy (EDS) mappings were carried out in a Scios 2 dual beam system (ThermofisherScientific) equipped with an EDS system (Oxford Instruments). Electron beam parameters of 10 kV energy and 1.6 nA beam current were implemented during investigations. Transmission electron microscopy (TEM) analyses were performed using a Titan G2 (ThermofisherScientific) operating at 300 kV. The tool has Cs corrected beam optics to reduce spherical aberration for ultra-high resolution imaging. A micro-Raman spectrometer (Renishaw) at an excitation wavelength of 532 nm and X-ray diffraction (XRD) using a D8 Discover diffractometry (Bruker) (K_αCu_ = 1.54 Å) were employed to study the vibrational modes and the crystalline quality of the MoS_2_/MoO_2_ heterostructures, respectively. X-ray photoelectron spectroscopy (XPS) analyses were carried out using a ThermofisherScientific K-alpha spectrometer and a PHI VersaProbe III scanning XPS microprobe to investigate the surface composition of our samples. The optical reflectance was measured using an UV–Vis-near IR spectrometer (JASCO V-670). Current-voltages (I–V) curves and transient photocurrents were measured using an photoelectrochemical device (Autolab PGSTAT204, Metrohm) coupled either with a solar simulator with Air Mass (AM) 1.5G filter (LOT Quantum Design, 100 mW cm^−2^) or with an LED module (LED Driver kit, Metrohm). The LEDs (450, 470, 505, 590, and 627 nm) used, have a low spectral dispersion and are calibrated with a photodiode to determine their actual power density (mW cm^−2^) received by the sample. To perform photo-electric measurements on our samples, we first exfoliate the MoS_2_/MoO_2_ nano-fibers and then transfer them onto a substrate with a circular interdigitated electrodes pattern^33^ with 10 µm spacing. The effective detection area of our sample is equal to 5 × 10^–3^ cm^2^.Atomic force microscopy (AFM) measurements were carried out in ambient conditions using a Dimension Icon system (Bruker, Santa Barbara, USA) in the peak force Kelvin probe force microscopy (PF-KPFM) method. Conductive Platinum coated silicon AFM probes (Spark150, NuNano, Bristol, UK), with a spring constant k = 20 ± 0.2 N/m, were used. The PF KPFM measurement enables the simultaneous characterization of the sample surface topography and surface potential variations.

## Results and discussion

**Figure 2:**
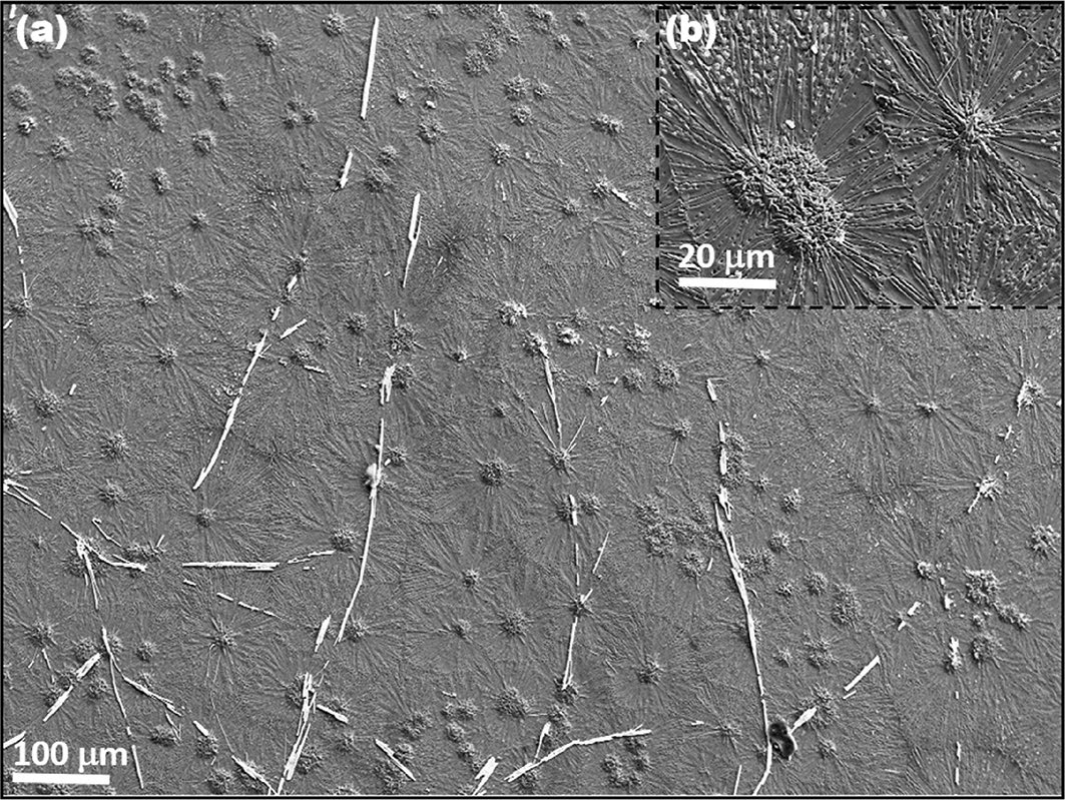
(**a**) Large view of the typical microstructure of as-grown MoS_2_/MoO_2_ heterostructures. (**b**) The inset shows a zoomed-in part of the image shown in (**a**), where the microfibers are seen to emerge from the central microflowers.

**Figure 3:**
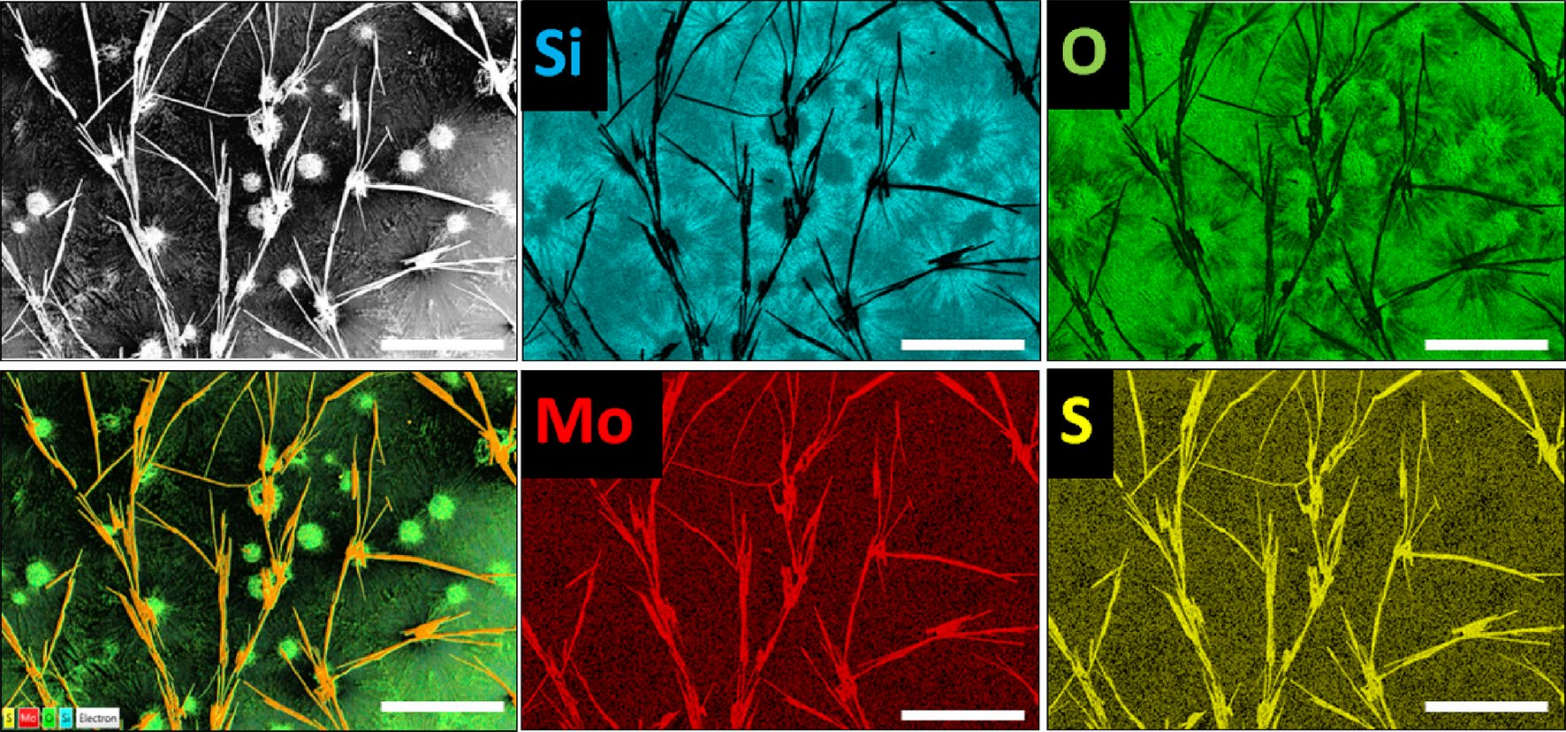
EDX mapping of MoS_2_/MoO_2_ heterostructure (scale bar = 100 µm).

The CVD grown MoS_2_/MoO_2_ heterostructure was first examined in SEM. The samples display a unique morphology consisting of microflowers attached to microfibers_,_ as shown in Fig. 2a. A close examination of these structures (see sections below) indicate that the microflowers mainly consist of MoO_2_ with an average diameter size of ~ 50 μm, and the microfibers correspond to MoS_2_ with a length reaching up to few hundreds of micrometers. Intermixing phases were also observed, especially at the boundaries, as it will be identified later by EDS, HRTEM and KPFM. The inset of Fig. 2b provides a closer look at the morphology of the fabricated MoS_2_/MoO_2_ heterostructures, showing MoO_2_ microflowers seemingly at the sites of MoS_2_ microfibers nucleation. This is well illustrated in the EDX elemental maps in Fig. 3. Individual elemental maps for Si, O, Mo, and S are separately shown, whereas their combined mapping was overlaid (color contrast) on the SEM image (bottom-left). The corresponding contrast provides clear indications that the microflowers are rich in oxygen whereas microfibers have a rather high sulfur content, which is signature of MoS_2_ compound.

**Figure 4:**
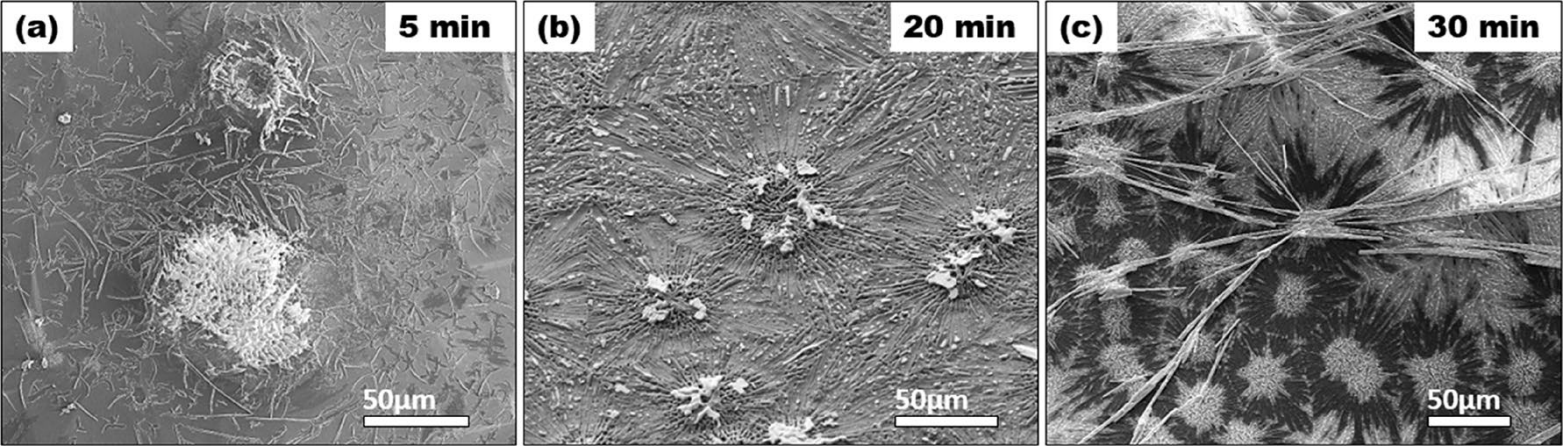
CVD reaction performed at different dwel times: (**a**) 5 min, secondary electron SEM image of nucleation sites of MoS_2_/MoO_2_ heterostructure, (**b**) 20 min secondary electron SEM image of MoS_2_/MoO_2_ heterostructure growth, (**c**) 30 min backscattered electron SEM image of well-settled MoS_2_/MoO_2_ heterostructure covering the entire substrate.

To examine the mechanism of MoS_2_/MoO_2_ heterostructure nucleation and growth, the CVD reaction was interrupted at different times while maintaining the same growth conditions. This allows monitoring the CVD reaction kenitics and analyzing the evolving compound miscrostructure. Figure 4. depicts the MoS_2_/MoO_2_ heterostructure after 5 min, 20 min and 30 min reaction time.

One can notice in Fig. 4a that there is first a nucleation sites, two in this micrograph, then random fibers are generated. 20 min later (Fig. 4b) several thicker fibers impinge from the nucleation sites and begin to make the connections between the nucleation sites. At last stage of the CVD reaction 30 min (Fig. 4c), the Si substrate is fully covered by very thick and long fibers as also shown in Fig. 3.

**Figure 5:**
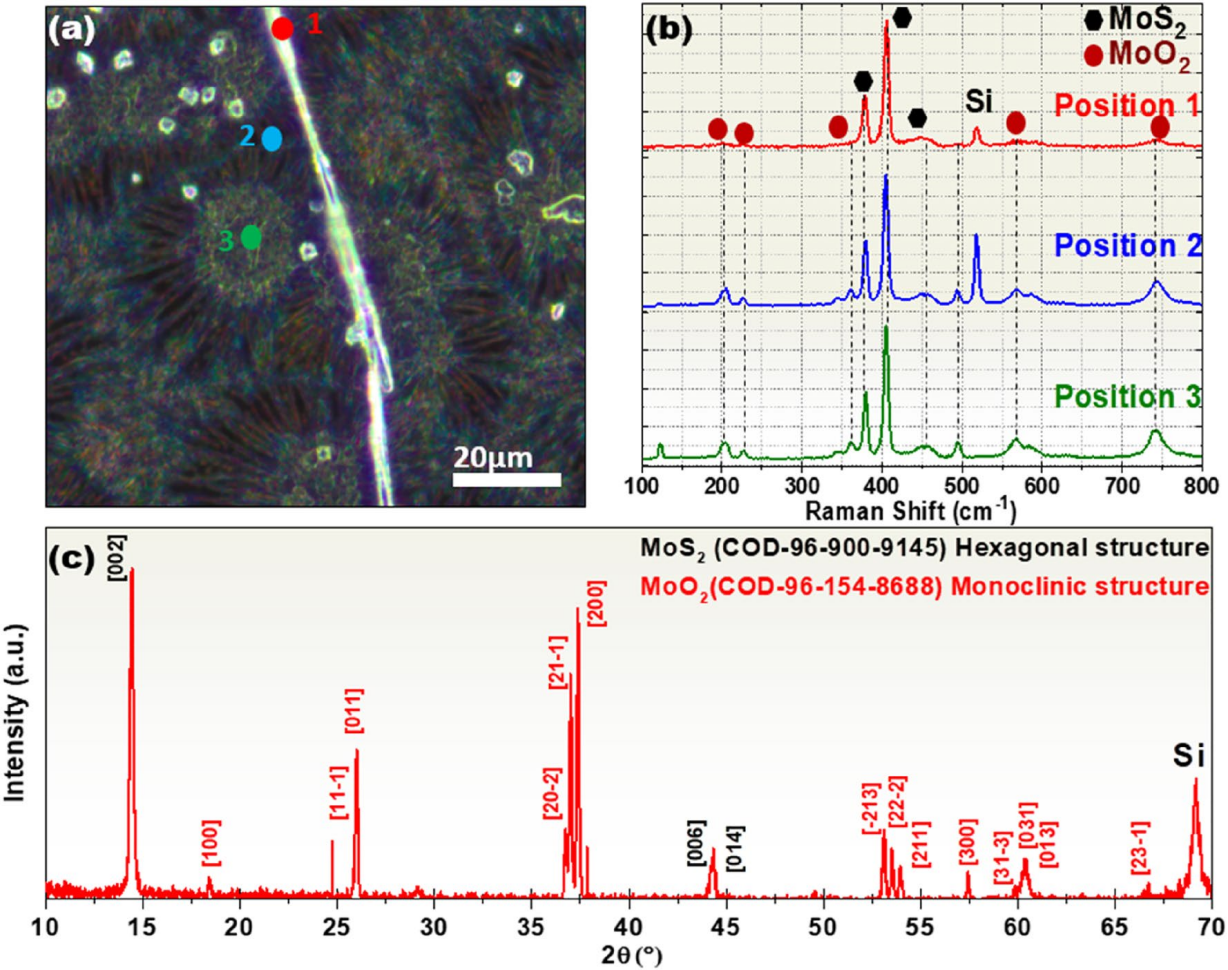
(**a**) Optical image showing the positions excited by the laser beam (532 nm) and their (**b**) respective Raman spectra of the MoS_2_/MoO_2_ heterostructures, (**c**) XRD diagram of the same.

The MoS_2_/MoO_2_ heterostructures were further characterized using Raman spectroscopy (laser excitation at 532 nm) and XRD diffraction. Figure 5a shows an optical image indicating the three locations on the sample excited by the laser beam. Figure 5b depicts their respective Raman spectra in the 100–800 cm^−1^. At the position 1, the vibrational modes are associated with known MoS_2_ peaks, i.e. E^1^_2g_ mode at 382 cm^−1^, A_1g_ mode at 406 cm^−1^^34^, and Si at 519 cm^−1^, indicating the good purity of MoS_2_ microfibers. Whereas for the position 2 and 3, corresponding to the microflowers core and edges, the spectra show additional vibrational modes translated by several peaks at 204, 226, 346, 363, 460, 494, 571, 589, and 742 cm^−1^ corresponding the MoO_2_ compound^32^. It was previously reported that the thermal deposition of MoO_3_ on Si substrates can lead to the formation of metallic MoO_2_ microflowers resulting from the thermal reduction of MoO_3_^35^. The Raman peaks at 204–494 cm^−1^ are due to the stretching modes of doubly coordinated oxygen (Mo–O–Mo)^36^, whereas the signals at 571, 589, and 742 cm^−1^ are due to terminal oxygen stretching modes (M=O)^37^. The formation of MoS_2_ and MoO_2_ phases is further supported by the XRD results. Indeed, the XRD diagram shown in Fig. 5b reveals the presence of three diffraction peaks (at 2θ = 14.41°, 29.11°, and 44.31°; labeled in black), which are ascribed respectively to the (002), (004) and (006) planes of the hexagonal phase MoS_2_. On the other hand, the multiple peaks located at 2θ = 18,43°, 25.85, 26°, 36.73°, 36.99°, 37.37°, 53.12°, 53.52°, 53.95°, 57.43°, 59.86°, 60.36°, 60.61°, and 68.25°, are attributed to the $\left(100\right)$, $(11\overline{1 })$, $\left(011\right)$, $(20\overline{2 })$, $(21\overline{1 })$, $\left(200\right)$, $(\overline{2 }13)$, $(22\overline{2 })$, $\left(211\right)$, $\left(300\right)$, $(31\overline{3 })$, $\left(031\right)$, $\left(013\right)$, and $(23\overline{1 })$ plans of MoO_2_ monoclinic structure, respectively.

**Figure 6:**
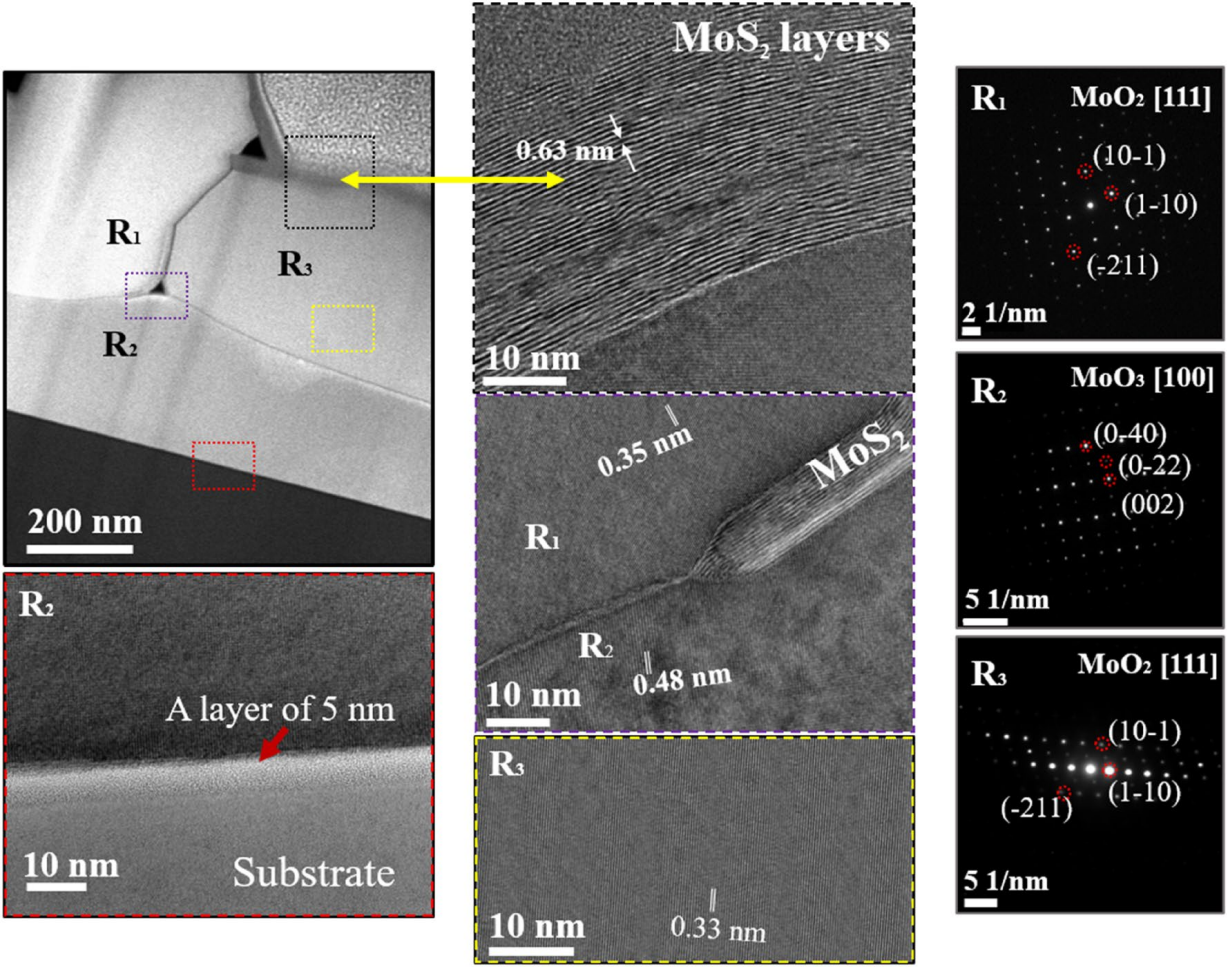
HRTEM images of the CVD-grown MoS_2_/MoO_2_ samples. (Top left): overview showing three regions, i.e. base of the MoO_2_ microflower, passivation substrate, and an impinging MoS_2_ fiber. (Bottom left): high magnification image of the substrate-grown sample interface. (Middle): crystal structures analyses for the 3 defined regions. (Right): corresponding diffraction patterns.

To further examine the microstructure of the MoS_2_/MoO_2_ heterostructure, cross-section samples were prepared (supplementary information) and HRTEM was performed at the MoO_2_ microflower and MoS_2_ microfiber interface to identify pure phases and intermixing sites, as shown in the top left image of Fig. 6. Three regions are identified as follows: region R1 corresponding to the base of the MoO_2_ microflower, as indicated by the central image showing the typical interplanar distance of 0.35 nm and the corresponding diffraction pattern given in the right image. Region R2 corresponding to the first fabricated layer exhibiting a MoO_3_ crystal structure underlined by the typical interplanar distance 0.48 nm and the corresponding diffraction pattern shown in the right images. This layer most likely acts as a passivation layer between the Si substrate and the MoS_2_/MoO_2_ heterostructure. Finally, region R3 corresponding to the nucleation site of the MoS_2_ microfiber, as indicated in the top middle image by the typical interplanar distance of 2H-MoS_2_ of 0.63 nm. In addition, R_3_ region reveals a thickness of ~ 32 nm, which corresponds to ~ 50 layers of 2H-MoS_2_. This is consistent with the crystalline structure of the MoS_2_ microfibers derived from XRD and Raman spectroscopy. More importantly, HRTEM shows that the MoS_2_ microfibers are intimately interconnected with the MoO_2_ microflowers. The electron diffraction analyses of the three regions indicate d-spacing of $(11\overline{1 })$, $(100)$, and $(011)$ planes corresponding to monoclinic m-MoO_2_ crystal. These results confirm the high crystalline quality of the MoO_2_ microflowers. Interestingly, the central image in Fig. 6 clearly reveals the co-presence of the three zone, indicating the heterostructure character of the MoS_2_ with both MoO_2_ and MoO_3_ on one side and a possible MoO_2_/MoO_3_ heterostructure on the other side. As the thickness of the MoO_3_ layer is negligible (5 nm: image bottom left) compared to microflowers/microfibers thickness, we assume that the general optical and photodetection behaviors are mainly due to the MoS_2_/MoO_2_ heterostructure.

**Figure 7:**
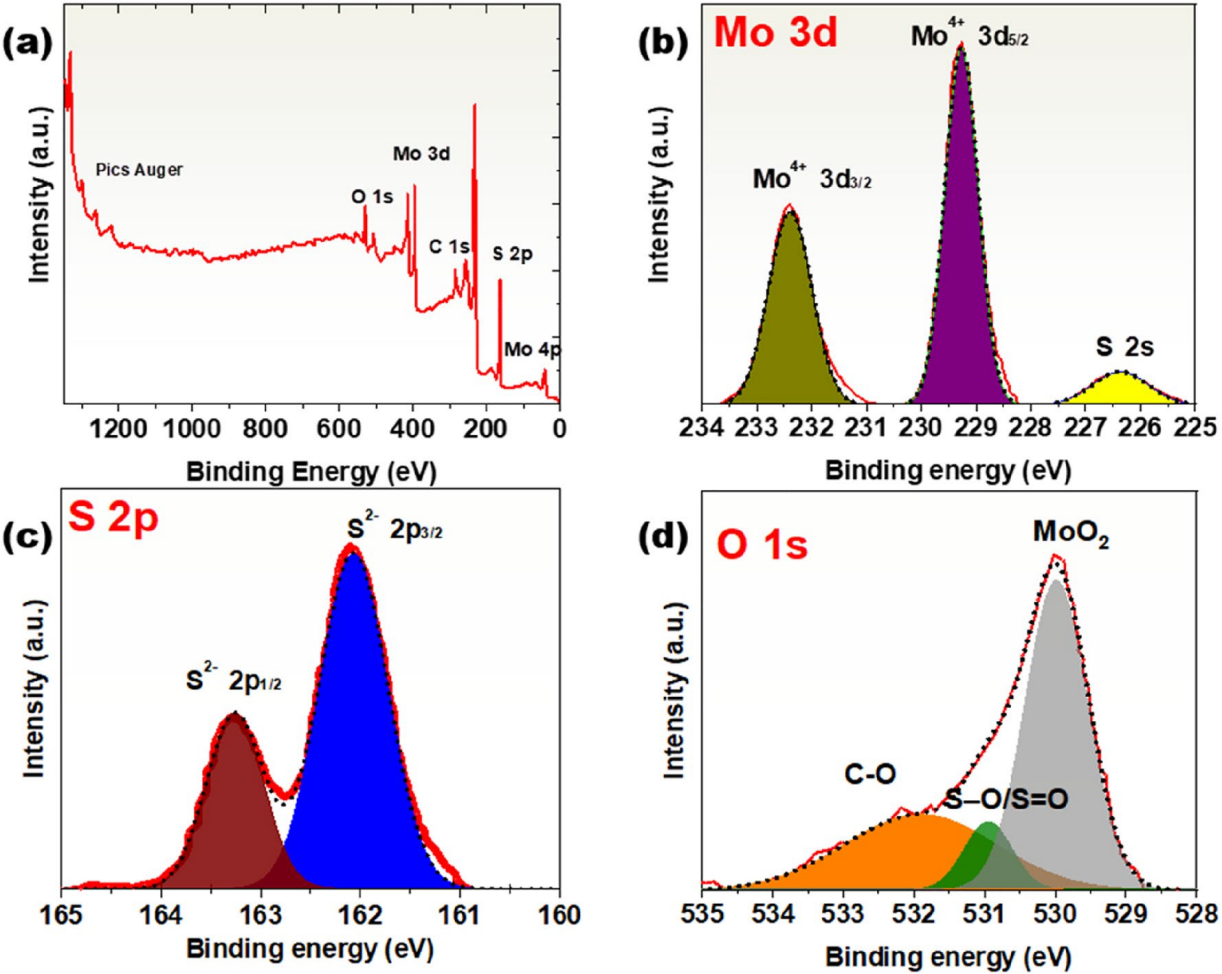
(**a**) X-ray photoelectron survey spectrum of the CVD grown MoS_2_/MoO_2_ heterostructures. (**b–d**) High-resolution spectra of the Mo 3d, S 2s, and (**c**) S 2p (**d**) O 1s core level peak region.

Figure 7a depicts the XPS survey spectrum of the CVD-grown MoS_2_/MoO_2_ samples. The high-resolution view of both Mo 3d and S 2s core peaks is shown in Fig. 7b. The peak appearing at 226.5 eV is a signature of the S 2s core level. The two peaks located at 229.3 and 232.4 eV are ascribed to the doublets Mo 3d_5/2_ and Mo 3d_3/2_, corresponding to the Mo^4+^ state in MoS_2_. Similarly, the S^2−^ doublet is observed in the S 2p spectra at 162.1 and 163.25 eV (Fig. 7c). The binding energies of O 1s are depicted in Fig. 7d. The peak around 530 eV corresponds to MoO_2_, whereas the peaks at 530.95 and 532 eV are attributed to S–O/S=O and C–O bonds, respectively.

**Figure 8:**
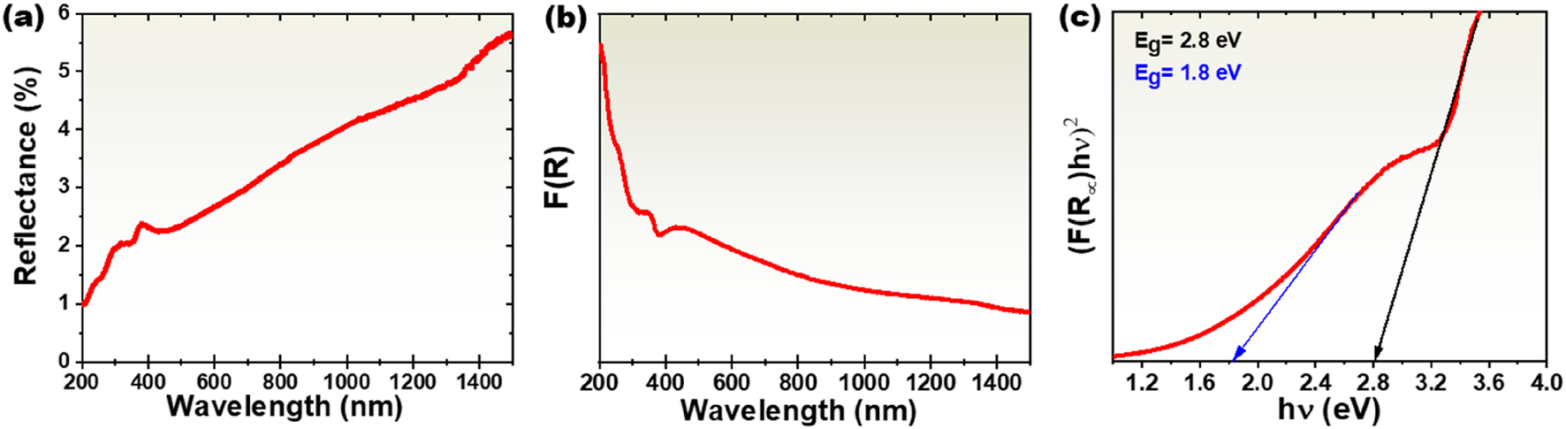
(**a**) Measured reflectance of the CVD-grown MoS_2_/MoO_2_ heterostructure samples. (**b**) Light absorbance as derived from the Kubelka–Munk function. (**c**) Bandgap estimation using Kubelka–Munk function plot.

Optical properties of the MoS_2_/MoO_2_ heterostructure samples were investigated by measuring their reflectance in the wavelength range of 200–1500 nm at room temperature. Figure 8a shows that the samples exhibit a very low reflectance over the entire investigated spectral range, increasing from 1% at 200 nm to only ~ 5.5% at 1500 nm. This very low reflectance is could be attributed to the change of MoS_2_ morphology in the presence of MoO_2_, as previously reported^38,39^. A weak reduction is observed on the reflectance spectrum around 430 nm attributed to the electron transitions, which occur in the optical band gap. The obtained overall reflectance values were used to evaluate the optical band gap using the Kubelka–Munk function (F(R)), plotted using light absorbance in Fig. 8b. The plot of (F(R)hν)^2^ versus photon energy (hν) and an approximation for direct band gaps of MoS_2_/MoO_2_ allowed extracting two potential values of direct bandgaps, namely E_g_ = 2.8 eV and E_g_ = 1.8 eV, as shown in Fig. 8c.

The band gap E_g_ = 2.8 eV could be ascribed to MoO_2_^18,40^, whereas E_g_ = 1.8 eV is consistent with the values quoted for direct bandgap of mono to few-layers of MoS_2_^41,42^. The presence of two band gap energies would enhance the probability for electrons to jump to both MoO_2_ and MoS_2_ conduction bands once excited by an external light source. This might have likely led to the higher broadband light absorption recorded during the optical absorption measurements. Taking advantage of the presence of band gap energies, Z-scheme electron excitation may be also activated. The consequence of such optical properties is discussed further in the photodetection measurement section.

**Figure 9:**
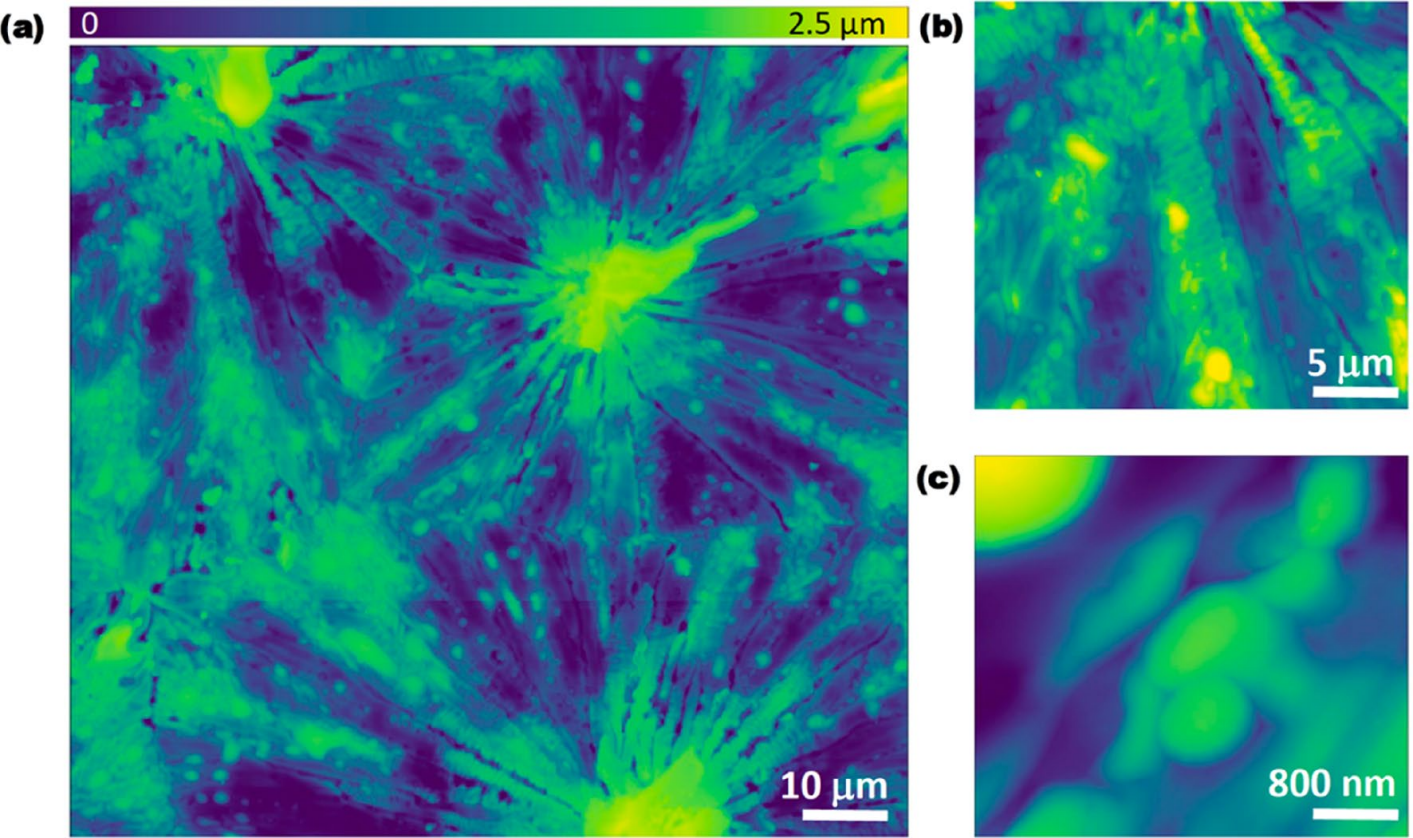
(**a**) AFM topography map (scan size: 80 µm^2^) showing details of the MoS_2_/MoO_2_ structure. (**b**) Area on the elongated structure in form of microfibers (scan size: 30 µm^2^) emanating from the central volcano structure. (**c**) A zoomed scan (scan size: 4 µm^2^) over an elongated petal structure showing the inclusion of multiple nanostructures.

KPFM was used to characterize the surface potential the MoS_2_/MoO_2_ heterostructure. Figure 9 shows topography maps acquired at different scan sizes. The flower-like structure previously observed in SEM is identified on the large scan are (80 µm × 80 µm) in Fig. 9a. Central volcano-like structures, distributed over the surface, from which elongated petal-like structures emanate in all directions forming microflower-like islands can be clearly observed.

**Figure 10:**
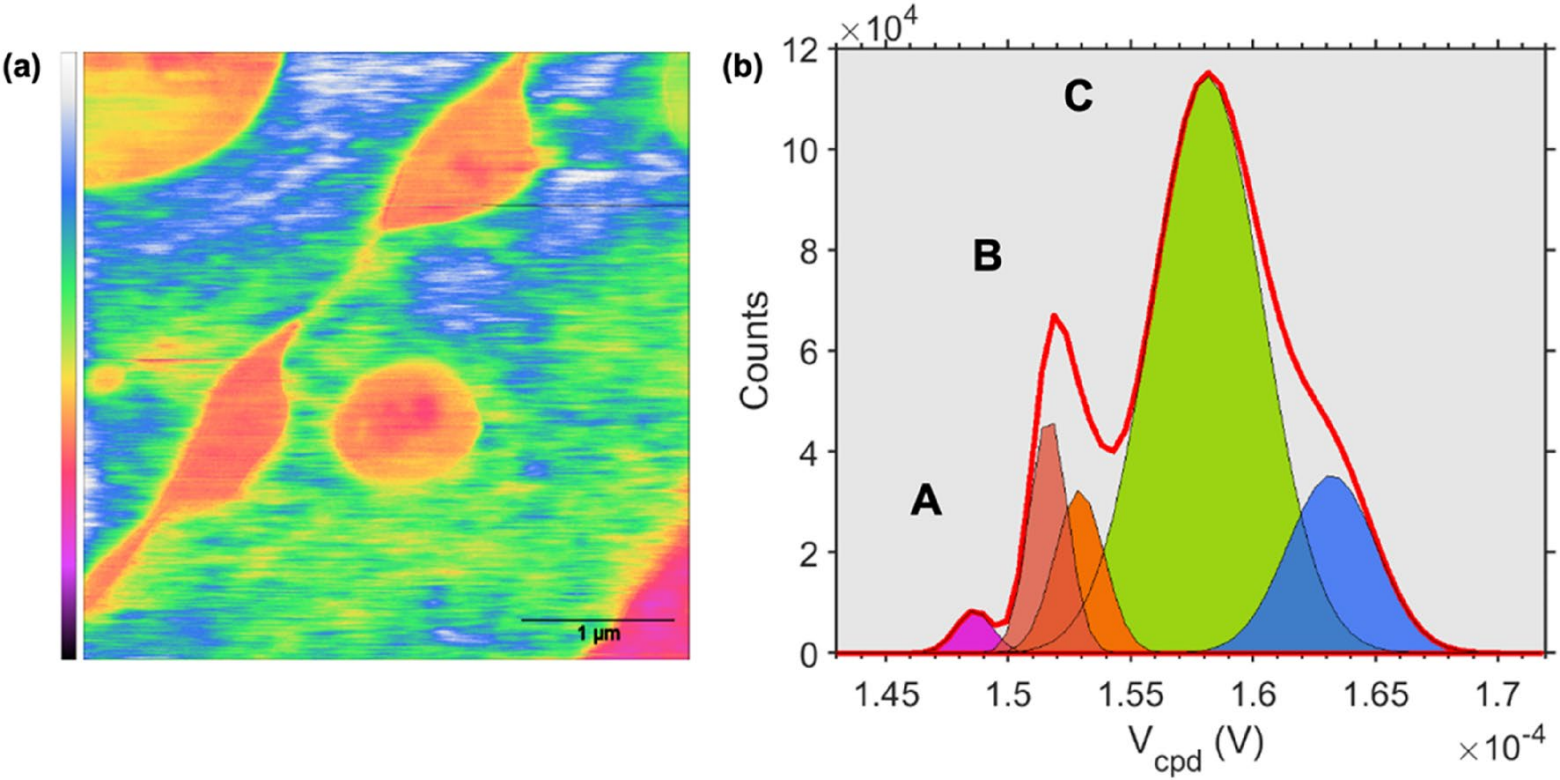
(**a**) Surface potential mapping acquired in the FM-KPFM mode on the same spot as in Fig. 8a. (**b**) Histograms of values extracted from the map shown in (**a**).

The large topography map reveals domain boundaries between islands, which points towards the growth dynamics dictated by surface energies and thermodynamic conditions. Fade dashed lines delimiting one island boundaries (lower right part of the image in Fig. 9a) were overlaid on the topography map to guide the eye. Interestingly, the magnified topography map (scan area: 30 µm × 30 µm) in Fig. 9b reveals the micromorphology of the elongated petal-like structures emanating from the center of the flower-like structure. Multiple microstructures intercalated in forms of inclusions are clearly observed. To further investigate the origin of this complex structural diversity, a spot with a coexisting mix of microstructures has been imaged (scan size: 4 µm × 4 µm), as shown in Fig. 9c. To investigate the local physical-chemically dependent characteristics of the coexisting structures, surface potential measurements were performed on the same spot in Fig. 9c. The surface potential (or contact potential difference, *V*_*cpd*_) stems from the differences in work functions between the AFM probe and the sample. It is an extreme surface-dependent property, highly sensitive to minute variations in the surface chemistry, electronic and crystallographic properties. As described in the experimental section. The AFM electrical measurements, shown here, were conducted in the PF-KPFM mode enabling simultaneous characterization of the surface adhesion to the AFM probe. Figure 10a shows the surface potential variations on the same spot as in Fig. 9c. The observed contrast indicates the coexistence of three different materials with different surface potential values. A comparison with the central image in Fig. 5, reveals a clear resemblance to the coexisting regions observed in HRTEM images. This strongly suggests a direct corroboration between the surface potential variations and that of the MoS_2_/MoO_2_ heterostructures. The histogram in Fig. 10b reveals three main peaks (A, B, and C) corresponding to the domains observed on the surface potential map (peak colors have been matched to the false color scale in Fig. 10a). The surface potential measurements provide a nanoscale electrical property signature, confirming the observation made above regarding the co-presence of three regions forming the heterostructure.

**Figure 11:**
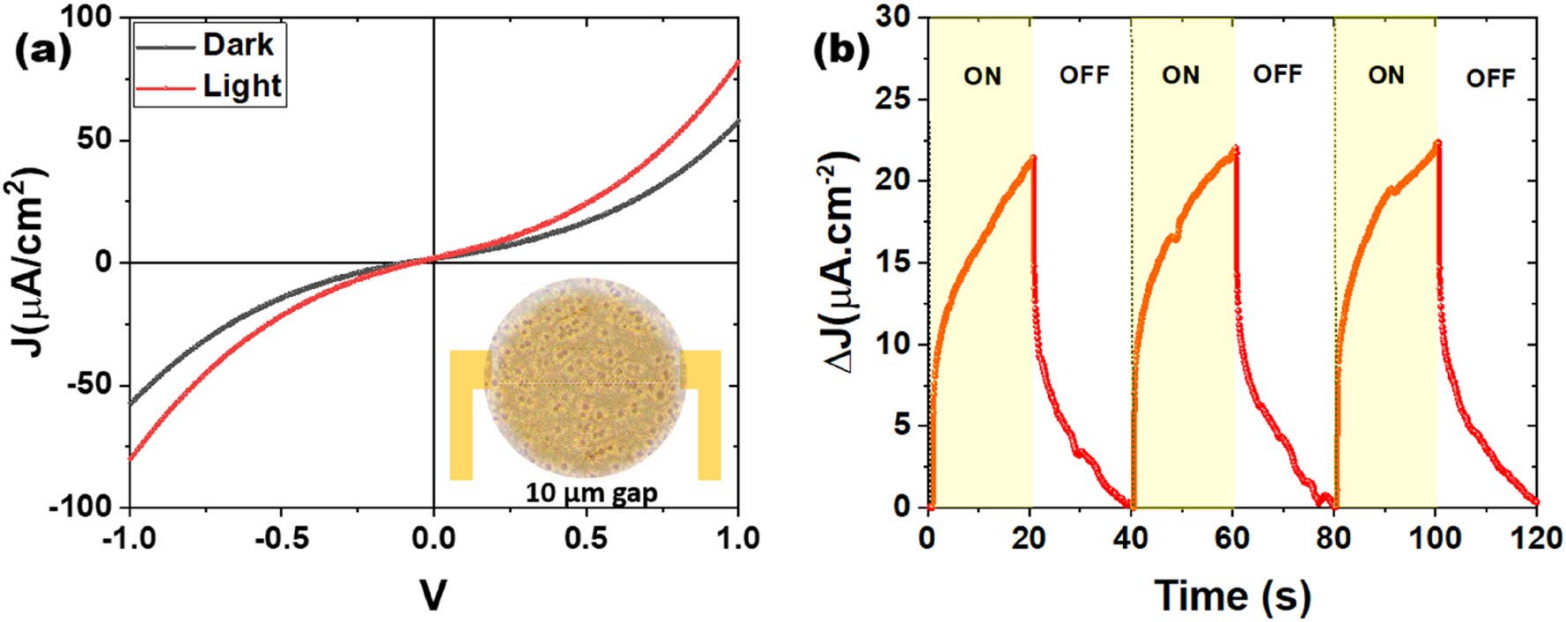
(**a**) J–V curves recorded under both dark and simulated sunlight conditions. (**b**) Transient photocurrent density response registered for 3 ON/OFF cycles (every 40 s) under standard sun illumination AM1.5 with an applied voltage V_bias_ = 1 V.

To investigate the photoelectric properties of our MoS_2_/MoO_2_ heterostructures, the samples were integrated into photoactive devices, and exposed to solar excitation using a solar simulator equivalent to one sun (i.e. 100 mW.cm^-2^ with AM 1.5G filter). Figure 11a shows J-V curves recorded under both dark (J_dark_) and solar excitation (J_light_). It is clearly seen that the J_light_ under sun irradiation is higher than its dark counterpart, confirming the photoactivity of our MoS_2_/MoO_2_ material. Figure 11b depicts the transient photocurrent density (ΔJ = J_light_ − J_dark_) response obtained at 1 V bias with successive ON/OFF cycles. A maximum photocurrent density is obtained after 20 s of sun exposure with a stable photocurrent density of 22 µA cm^−2^ indicating the high stable photoactivity of the heterostructure. On the J-V curve, a symmetry in the ON/OFF cycles with respect to the origin is observed, suggesting the presence of an ohmic contact between the heterostructure and the gold electrodes, in accordance with previously reported works. This reveals a good electrons’ injection at the MoS_2_/Au interface compatible with the presence of an ohmic contact^12,13^, which can be partially explained by the good chemical affinity between the gold and sulfur atoms leading to very weak injection barrier. Nonetheless, J-V curves exhibit a linear variation for low voltages followed by a curvature for higher voltages. Assuming the contacts are indeed ohmic, such curvature is probably due to space-charge-limited current effects^43^. In addition, Fig. 11b shows that the photocurrent undergoes a slow increase under standard sun illumination, and conversely, it shows a slow decay under darkness condition. The observed slow kinetic behavior is an indicator of carrier trapping within the heterostructure under illumination followed by a thermal detrapping of carriers manifested by the slow decay^43^.

**Figure 12:**
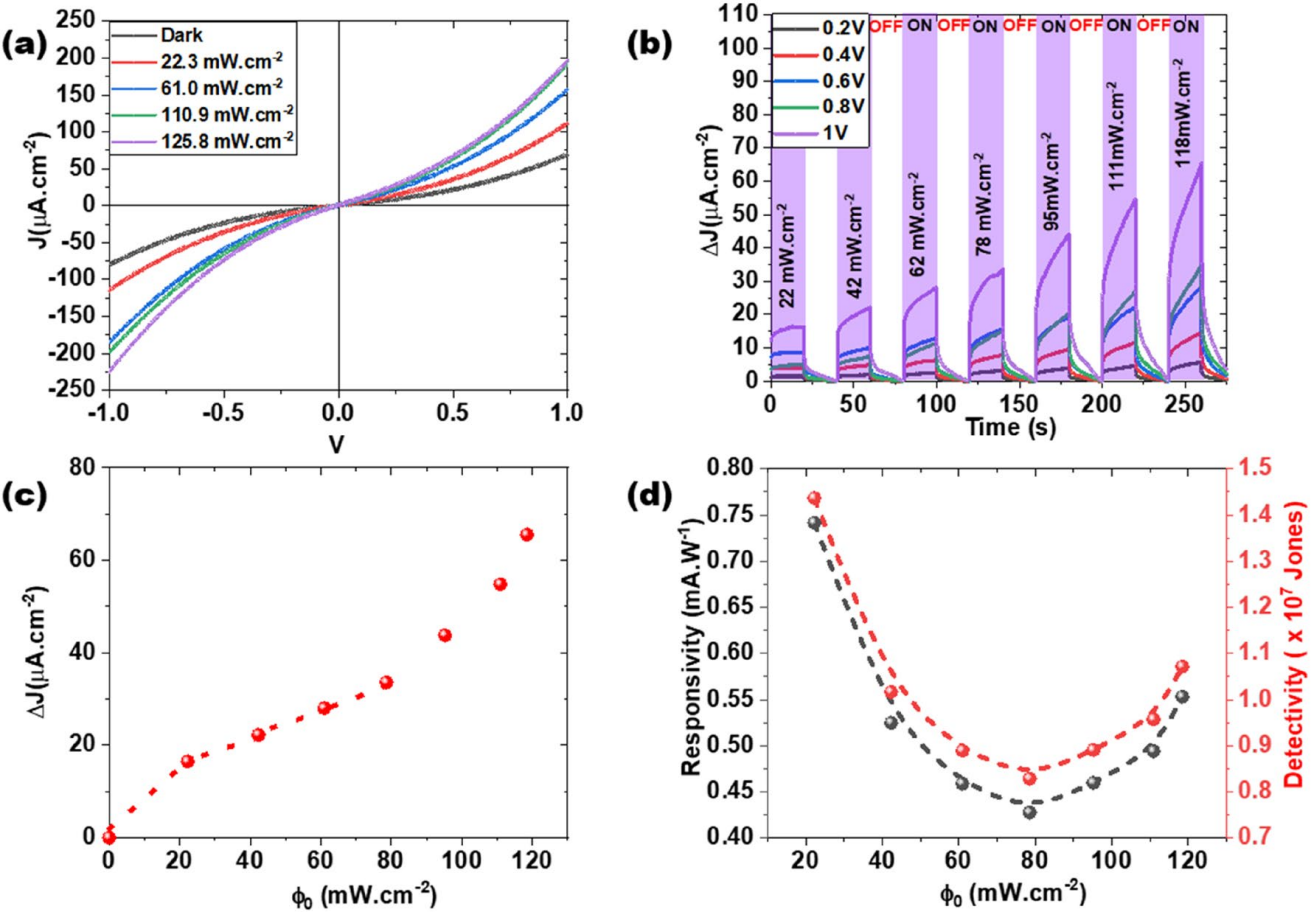
(**a**) J–V curves recorded in the dark and under different excitations light intensities at λ = 450 nm. (**b**) Transient photocurrent density for various ON/OFF cycles (40 s each) as a function of light intensity at λ = 450 nm. (**c**) Photocurrent density variation as a function of the light intensity of the blue laser at 1 V bias. (**d**) Responsivity and detectivity change with the incident blue light intensity at 1 V bias.

According to the absorbance spectrum obtained (see Fig. 8a,b) the MoS_2_/MoO_2_ heterostructure exhibits high absorbance capacity in the blue region. To elucidate this optical behavior, we carried photoresponse investigations of our MoS_2_/MoO_2_-based device under 450 nm laser excitation. Figure 12a shows the typical J-V curves recorded in dark and under illumination at variable laser power densities. Our results show that the photogenerated current density under blue light excitation is continuously increasing with increasing power density in the full range between −1 and 1 V. At a power density of 125 mW cm^−2^, the photogenerated current density is five times higher than the one recorded in dark. Hence, the high optical absorption of our MoS_2_/MoO_2_ heterostructure is translated to higher photoconversion capacity. The stability and performance of our heterostructure-based photodetector were then further investigated. Figure 12b illustrates the transient photocurrent response steps during light ON/OFF cycles at increasing light power densities ${\phi }_{0}$. Results clearly show that the MoS_2_/MoO_2_ heterostructure is stable over time and its photogenerated current is continuously increasing with increasing light power density and/or applied bias. To examine the behavior of the photocurrent change induced by the variation of the incident light intensity, the ${\phi }_{0}$ dependence of the photocurrent is depicted in Fig. 12c. Two main behaviors can be identified as follows: (1) For ${\phi }_{0}<$ 80 mW cm^−2^ the photocurrent density (ΔJ) follows the classical power law ΔJ = αΦ_0_^n^, where α is a wavelength-dependent constant and n ≤ 1 is a constant. (2) For ${\phi }_{0}\ge$ 80 mW cm^−2^ there is a clear change in the slope of the photocurrent a steep increase of the photocurrent with increasing ${\phi }_{0}$. The obtained photocurrent was subsequently used to determine the responsivity and detectivity of our MoS_2_/MoO_2_-based photodetector according to the following equations:

$$R=\frac{{I}_{p}}{{\phi }_{0}A}\,\,\,\mathrm{ and }\,\,\, {D}^{*}=\frac{R\sqrt{A}}{\sqrt{2q{I}_{Dark}}},$$

where I_p_ is the generated photocurrent (i.e. difference between the current under illumination and dark current), A is the effective irradiated area, q is the electron charge and I_Dark_ is the dark current.

The obtained responsivity and detectivity as function of the light power densities ${\phi }_{0}$ are depicted in Fig. 12d. Our MoS_2_/MoO_2_-based photodetector exhibits maximum values for both responsivity and detectivity at 0.75 mA W^−1^ and 1.45 × 10^7^ Jones, respectively, achieved at very low blue light intensity excitation of 20 mW cm^−2^. It is woth noting that high optoelectronic performances based on MoS_2_ were reported^44^, however, the fabrication routes cited in this study are tedious, consisting of several fabrication steps. Additionally their photodection responses were achieved on very small active area (~ 10^–7^ cm^2^) and at high applied bias (~ 20 V) compared to our measurements. Generally, our findings concur with the recently reported data as summarized in Table 1.

**Table 1:** Comparison of MoS_2_ heterostructures based photodetectors.

Heterostructure	Processing	Excitation (nm)	Bias (V)	R (mA/W)	D* (× 10^7^ Jones)	Ref
MoS_2_/ZnS	Hydrothermal	554	1	0.17	–	^10^
MoS_2_/SnS	Magnetron sputtering	473	1	2.4	5.7	^45^
MoS_2_/MoTe_2_	Mechanical exfoliation	532	0	111	14.8	^46^
MoS_2_/MoO_x_	CVD	638	10	10	600	^47^
MoS_2_/MoO_x_	CVD	405	10	1090	2.8 × 10^4^	^47^
MoS_2_/GaN	CVD	460	20	25	56	^14^
MoS_2_/MoO_3_	Chemical exfoliation	405	5	0.134	–	^48^
MoS_2_/MoO_2_	CVD	450	1	0.75	1.45	This work

In this table, we have summarized MoS_2_/MoO_2_ based photodetector performances while providing precision on the fabrication technique used, the excitation energy and the bias voltage. One can notice that our results is comparable to reported data, which indicates the ability of the one-step CVD process to compete with other fabrication techniques.

Nonetheless, Fig. 12d shows that as the incident ${\phi }_{0}$increases, both responsivity and detectivity are decreasing until reaching their minimum values at ${\phi }_{0}=$80 mW cm^−2^. Surprisingly, this decrease was followed by a slight augmentation in both the responsivity and detectivity values for higher incident light intensities. This could be ascribed to the presence of several photoactive layers within the MoS_2_/MoO_2_ heterostructures as suggested by both HRTEM and KPFM surface potential images. The MoO_2_ photoactive layer anchored to the MoS_2_ layer contributes to the photocurrent generated when the power density of the light is high enough to pass through the oxide layer. Thus, up to 80 mW cm^−2^, we observe a power law (e.g. Fig. 12c), beyond which we observe the contribution of the MoO_2_/MoS_2_ heterojunction.

**Figure 13:**
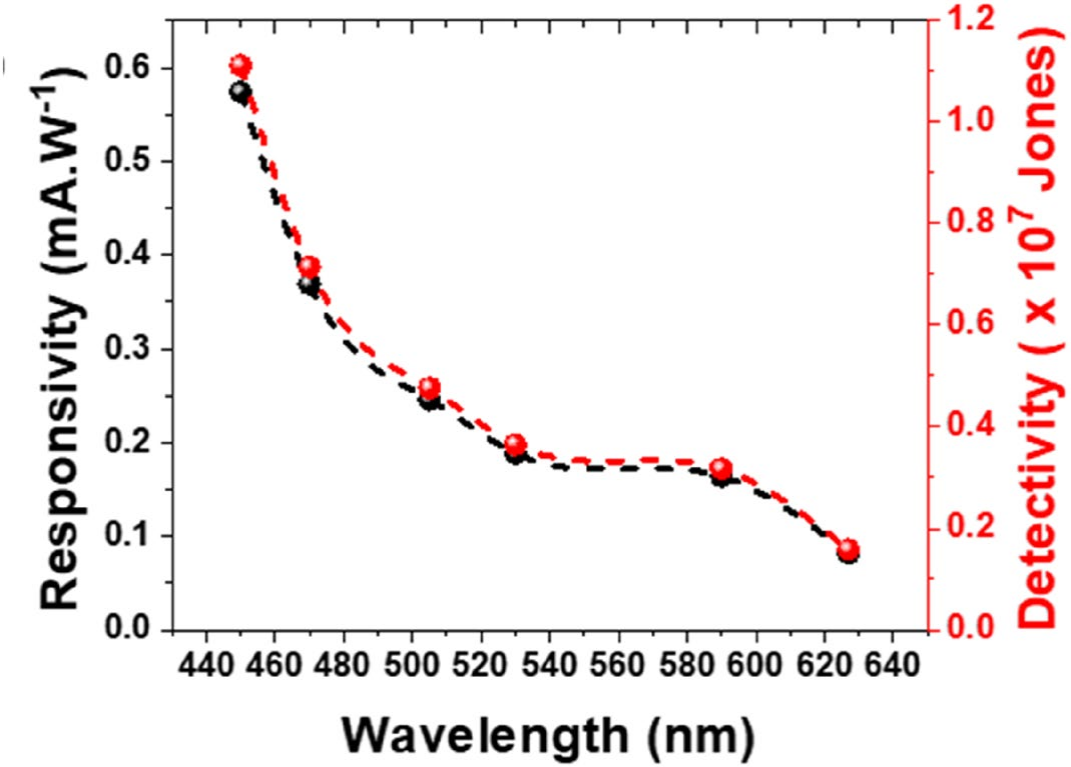
Wavelength dependence of the responsivity and detectivity of the MoS_2_/MoO_2_ based photodetector, obtained at 40 mW cm^−2^ light power density and 1 V bias.

Furthermore, The radiation-responsivity and detectivity of the MoS_2_/MoO_2_ heterostructure was studied using various excitation wavelengths at 40 mW.cm^-2^ light power density and 1 V bias as shown in Fig. 13.

The heterostructure photodetection performance is validate for the entire visible spectral range. Nonetheless, it is higher for shorter wavelengths, which is in good agreement with the aforementioned optical measurements. Indeed, the heterostructure is six times more responsive at 450 nm than at 630 nm wavelength. Finally, it is worth noting that the photoresponse performances are measured on the entire sample (microflowers and microfibers). We believe that the photoresponse could be improved if it is recorded using the sole microfibers. Hence different techniques could be put in place to isolate one or few microfibers using mechanical and/or chemical exfoliation or nanofabrication using FIB-SEM to allow developing these microfibers onto photoconductive device.

## Conclusion

In summary, complex MoS_2_/MoO_2_ heterostructures were successfully synthesized using a facile one-step CVD process. The synthesized heterostructures consist mainly of MoO_2_ microflowers of few 10 s microns in diameter, from which MoS_2_ microfibers up to 100 s microns-long emanate in all directions. X-ray and electron diffraction techniques have revealed that the crystalline nature of MoS_2_ and MoO_2_ in the respective hexagonal 2H-MoS_2_ and monoclinic m-MoO_2_ structures. These MoS_2_/MoO_2_ heterostructures were found to exhibit high broadband optical absorption over the entire 200–1500 nm spectral range. This high optical performance is ascribed to the presence of two bandgap energy values measured at 1.8 and 2.8 eV, consistent with those quoted for MoS_2_ and MoO_2_, respectively. The strong optical absorption was exploited by integrating the heterostructure samples into functional photodetectors, and interestingly found to exhibit high photoresponsive over the 450—630 nm range. The highest responsivity and detectivity values of 0.75 mA W^−2^and 1.45 × 10^7^ Jones, respectively, were obtained under the blue light excitation at very low light illumination of 20 mW cm^−2^. These results highlight the potential of these CVD-grown MoS_2_/MoO_2_ heterostructures for strong broadband light harvesting and photodetection applications.

## Supplementary Information

Supplementary Figure 1.

## Data Availability

All data supporting this work are available upon request from the corresponding author M. Jouiad.
