# Supplementary material for: Broadband photodetection using one-step CVD-fabricated MoS2/MoO2 microflower/microfiber heterostructures
Source: Sci Rep. 2022 Dec 21;12:22096. doi: 10.1038/s41598-022-26185-z (PMC9772214; doi:10.1038/s41598-022-26185-z)
Supplement: Supplementary file 1 — Supplementary Figure 1. [file 41598_2022_26185_MOESM1_ESM.docx]

**Supplementary information**

**Broadband photodetection using one-step CVD-fabricated MoS_2_/MoO_2_ microflower/microfiber heterostructures**

D. Mouloua^1,2^, NS. Rajput^3^, S. Saitzek^4^, K. Kaja^5^, K. Hoummada^6^, M. El Marssi^1^,

M. A. El Khakani^2*^, M. Jouiad^1*^

*^1^Laboratory of Physics of Condensed Matter, University of Picardie Jules Verne, Scientific Pole, 33 rue Saint-Leu, 80039 Amiens Cedex 1, France*

*^2^Institut National de la Recherche Scientifique, Centre-Énergie, Matériaux et Télécommunications, 1650, Blvd, Lionel–Boulet, Varennes, QC J3X-1S2, Canada*

*^3^Advanced Materials Research Center, Technology Innovation Institute, P.O. Box 9639, Abu Dhabi, United Arab Emirates*

*^4^ Univ. Artois, CNRS, Centrale Lille, Univ. Lille, UMR 8181, Unité de Catalyse et Chimie du Solide (UCCS), F 62300 Lens, France*

*^5^Laboratoire National de métrologie et d’essais (LNE), 29 av. Roger Hannequin, 78197 Trappes, France*

*^6^Aix Marseille Univ, CNRS, Université de Toulon, IM2NP, 13397, Marseille, France*

*Corresponding authors: m.a.elkhakani@inrs.ca; mustapha.jouiad@u-picardie.fr


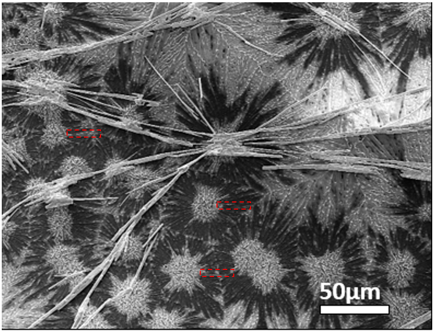


**Figure 1**: SEM image of MoS_2_/MoO_2_ heterostructure: red dashed boxes indicate the typical positions where the TEM lamellas are prepared using FIB-SEM conventional method.
